# Recent insights into luteolin and its biological and pharmacological activities

**DOI:** 10.17179/excli2024-7168

**Published:** 2024-05-16

**Authors:** Priscilla Nadalin, Jae Kwang Kim, Sang Un Park

**Affiliations:** 1 Department of Crop Science, Chungnam National University, 99 Daehak-ro, Yuseong-gu, Daejeon, 34134, Korea; 2 Division of Life Sciences and Convergence Research Center for Insect Vectors, College of Life Sciences and Bioengineering, Incheon National University, Incheon 22012, Korea; 3 Department of Smart Agricultural Systems, Graduate School, Chungnam National University, Daejeon 34134, Republic of Korea; 4 Department of Bio-AI Convergence, Chungnam National University, 99 Daehak-ro, Daejeon 34134, Republic of Korea

## ⁯

Luteolin (LUT), or 3',4',5,7-tetrahydroxyflavone, is a flavonoid generally found in glycosylated form in a wide range of plants such as medicinal herbs, fruits and vegetables (Arampatzis et al., 2023[2]). Chinese traditional medicine makes extensive use of LUT to treat numerous conditions, particularly inflammatory disorders, hypertension, and cancer (Lin et al., 2008[30]). According to IUPAC chemical nomenclature, LUT is named 2-(3,4-dihydroxyphenyl)-5,7-dihydroxy-4H-chromen-4-one. This compound was first isolated in its pure form in 1829 by a French chemist Michel Eugène Chevreul (Jain and Tiwari, 2020[19]). 

LUT exerts a range of beneficial effects on human health, with the following being reported in many studies: antiallergic, anti-inflammatory, antidiabetic, neuroprotective, and anticancer. Due to their chemical nature, LUT and its glycosides also display antioxidant properties, scavenging free radicals derived from oxidation and chelating metal ions (Cai et al., 1997[3]; Choi et al., 2007[8]; Muruganathan et al., 2022[33]). In recent years, LUT has attracted much attention from the pharmaceutical, food, and cosmetic industries for its plethora of biological and pharmacological activities. Herein, we present a summary of recent key studies performed to evaluate the biological and pharmacological activities of LUT (Table 1(Tab. 1); References in Table 1: Aljohani et al., 2023[1]; Arampatzis et al., 2023[2]; Chang et al., 2023[4]; Chen et al., 2022[6], 2023[5]; Cheng et al., 2022[7]; Ding et al., 2023[9]; Dong et al., 2023[10]; Eddy et al., 2024[11]; Fu et al., 2024[12]; Guo et al., 2024[13]; Han et al., 2022[14]; Hao et al., 2023[15]; He et al., 2023[16]; Huang et al., 2023[17][18]; Jang et al., 2022[20]; Ji et al., 2022[21]; Jia et al., 2023[22]; Jiang et al., 2022[23]; Kahksha et al., 2023[24]; Kariu et al., 2023[25]; Kim et al., 2023[26]; Li et al., 2022[27][28], 2023[29]; Liu et al., 2023[31]; Mousavi et al., 2022[32]; Nishiguchi et al., 2024[34]; Pan et al., 2022[35]; Qi et al., 2022[36]; Qiao et al., 2023[37]; Qin et al., 2022[38]; Ramadan et al., 2023[39]; Ren et al., 2024[40]; Rudin et al., 2023[41]; Song et al., 2022[42]; Sudhakaran et al., 2023[43][44]; Sur and Lee, 2022[45]; Tráj et al., 2023[46]; Wang et al., 2023[47]; Wen et al., 2024[48]; Xia et al., 2024[49]; Xie et al., 2022[50]; Xu et al., 2023[51]; Xue et al., 2023[52]; Yajie et al., 2023[53]; Yang et al., 2023[54]; Ye et al., 2023[55]; Yoon et al., 2023[56]; Yuan et al., 2023[57]; Zaki et al., 2023[58]; Zhang et al., 2023[59]; Zheng et al., 2023[60]; Zhou et al., 2022[61]; Zhu et al., 2022[62]).

| **Key findings** | **Reference** |
|---|---|
| LUT has proved beneficial in treating excessive intestinal motility and diarrhea via regulation of the nuclear factor erythroid 2-related factor 2 (Nrf2) signaling pathway, thus successfully alleviating oxidative damage to the colon. | Xia et al., 2024 |
| This work investigates the potential targets and molecular mechanism through which LUT ameliorates fumonisin B1-induced intestinal inflammatory injury. The results reveal that LUT could be a novel alternative antibiotic to prevent inflammatory injury in the intestines. | Wen et al., 2024 |
| LUT could be a promising future therapy for Parkinson's Disease, as it has proved able to prevent endoplasmic reticulum (ER) stress via the activation of the ubiquitin ligase 3-hydroxy-3-methylglutaryl-coenzyme A reductase degradation 1 (HRD1) and the suppressor/enhancer lin-12-like (SEL1L). | Nishiguchi et al., 2024 |
| LUT could be a novel therapy and protective compound for alleviating cytotoxicity in neurodegenerative pathologies such as Huntington's disease. | Ramadan et al., 2023 |
| *Lonicera japonica* extract may treat lipopolysaccharide-induced acute injury and inflammation in mice and BEAS-2B due to its LUT content, which triggers suppression of the nuclear factor kappa B (NF-κB) signaling pathway. | Jia et al., 2023 |
| LUT inhibits the ROS/phosphoinositide 3-kinase (PI3K)/v-akt murine thymoma viral oncogene (AKT) pathway, hence preventing mesothelial-mesenchymal transition and lowering postoperative abdominal adhesion (PAA). As such, LUT could be used to treat PAA. | Ren et al., 2024 |
| LUT has proved effective in alleviating acquired sensorineural hearing loss (SNHL)-related auditory cell apoptosis by acting on the JAK/STAT pathway, thereby providing a possible new tool to treat acquired SNHL. | Guo et al., 2024 |
| Administration of LUT resulted in relevantly lower NF-κB activation, and reduced expression of reactive oxygen species (ROS), superoxide, interleukin-6 (IL-6) and endothelin-1 in endothelial cells. The phosphorylation of NF-κB in human placental explants also appeared considerably diminished. These data show LUT's ability to inhibit pathways leading to the occurrence of preeclampsia (PE). For this reason, LUT should be investigated further to better understand its potential in treating PE. | Eddy et al., 2024 |
| LUT possesses anti-inflammatory properties that have proved valuable in combating periodontal disease and restoring damaged bone tissue. | Arampatzis et al., 2023 |
| LUT may be a safe choice and useful antiplatelet agent target for GPVI. This study identified a novel mechanism (diminished oxidative stress) for the effectiveness of LUT as an antiplatelet agent. | Ye et al., 2023 |
| LUT can prevent glioma cell proliferation, as well as cellular invasion and migration and promote both the apoptosis of glioma cells and the expression of apoptosis-inducing proteins, which makes LUT a suitable candidate treatment in the fight against glioma. | Yuan et al., 2023 |
| This study highlights LUT's protective activity against cigarette smoke-linked damage in a zebrafish model. | Sudhakaran et al., 2023b |
| LUT induces ferroptosis in prostate cancer cells by promoting nuclear translocation of the transcription factor EB and improving ferritinophagy. | Fu et al., 2024 |
| LUT acts in synergy with osimertinib to overcome acquired resistance to osimertinib induced by the mesenchymal-epithelial transition (MET) factor amplification and overactivation: the implicated mechanism is inhibition of the hepatocyte growth factor-MET-Akt pathway. These findings suggest that the combined action of LUT and osimertinib could be effective in treating non-small cell lung cancer in patients affected by osimertinib resistance. | Huang et al., 2023b |
| LUT can ameliorate dextran sulphate sodium-induced colitis, counteract IKKα/β, inhibit NF-κB signaling and prevent macrophage activation and migration. Hence, LUT could become a future promising treatment for colitis. | Xue et al., 2023 |

| **Key findings** | **Reference** |
|---|---|
| LUT can bind to the severe acute respiratory syndrome coronavirus 2 (SARS-CoV-2) major protease 3CL and mitogen-activated protein kinase 1, thus revealing its anti-SARS-CoV-2 potential. | Wang et al., 2023 |
| LUT suppresses planktonic growth and biofilm production in *Porphyromonas gingivalis*. In addition, orally administered LUT has proved effective in reducing maxillary alveolar bone resorption (ABR) in a mouse model of *P. gingivalis*-induced periodontitis. As such, LUT could become a novel treatment against *P. gingivalis* by blocking the bacterium's growth, biofilm formation, and ABR in the buccal cavity. | Kariu et al., 2023 |
| This study's results highlight LUT's anti-biofilm activity and provide a possible framework for improved treatment strategies against virulence factors of pathogens inducing biofilm production in the oral cavity. | Rudin et al., 2023 |
| LUT peracetate, gossypol and gossypolone have proved able to suppress immune complex-linked neutrophil adhesion and oxidative burst *in vitro*. Additionally, LUT peracetate and gossypolone inhibited anti-COL7 IgG-linked dermal-epidermal separation in an epidermolysis bullosa acquisita *ex vivo* model. | Yang et al., 2023 |
| LUT cooperates with erastin, causing colon cancer cells to be vulnerable to ferroptosis. The implicated mechanism is inhibition of glutathione peroxidase 4 expression induced by the hypermethylated in cancer 1 gene. Hence, these results suggest that LUT could be employed in future colon cancer treatments. | Zheng et al., 2023 |
| LUT can protect zebrafish from photodamage caused by UV-B via DNA-protective, antioxidant and anti-inflammatory activities. | Sudhakaran et al., 2023a |
| LUT successfully ameliorates cognitive deficits caused by triple-transgenic Alzheimer's disease (AD) in a mouse model, also suppressing β-amyloid-induced oxidative stress, mitochondrial impaired activity and neuronal apoptosis due to its peroxisome proliferator-activated receptor γ (PPARγ)-dependent mechanism of action. As such, LUT could become a novel AD treatment. | He et al., 2023 |
| This study shows the potential of LUT as a future antibiofilm agent targeting drug-resistant *Staphylococcus aureus* occurring in dairy products and goat farm environments. | Liu et al., 2023 |
| LUT showed greater antitumour activity than carboplatin, but a similar activity to that of norcantharidin in an *in vitro* system obtained from a patient-derived organoids (PDO) system. LUT also showed different extents of antitumor activity on PDO, hence suggesting that PDO could become a valid preclinical drug screening tool for personalized treatment. | Hao et al., 2023 |
| LUT can ameliorate hyperlipidemia-induced cardiac tissue injury in Sprague-Dawley rats, hence contributing to the development of novel therapeutic approaches to address cardiovascular disease progression. | Dong et al., 2023 |
| LUT can alleviate inflammation-induced acute lung injury by partially improving transepithelial sodium transport via the JAK/STAT pathway. Hence LUT could become a novel therapeutic agent for edematous lung diseases. | Chen et al., 2023 |
| LUT exerts its antineoplastic activity against Ehrlich solid carcinoma by diminishing the size and weight of the tumor while also enhancing muscle cell structure. It exerts its mechanisms by inhibiting Wnt, β-catenin, and SMAD4, which results in diminished proliferation and differentiation of cancerous cells. Furthermore, LUT induces overexpression of E-cadherin, resulting in decreased tumor cell invasion and metastasis. | Aljohani et al., 2023 |
| Lupus nephritis (LN) is an aggravation of systemic *lupus erythematosus* and a principal cause of mortality. LUT could be a possible therapy to prevent and treat LN via inhibition of hypoxia-inducible factor 1α (HIF-1α) expression in macrophages. | Ding et al., 2023 |
| LUT possesses anti-fibrotic activity in a laser-induced mouse model. It acts through suppression of the MET of retinal pigment epithelial cells, via deactivation of Smad2/3 and YAP signaling. As such, LUT could become a potential natural approach to prevent and treat subretinal fibrosis and fibrosis-related disorders. | Zhang et al., 2023 |

EXCLI Journal 2024;23:787-794 – ISSN 1611-2156
Received: March 12, 2024, accepted: April 23, 2024, published: May 16, 2024

| **Key findings** | **Reference** |
|---|---|
| A low concentration of LUT can protect liver cells from an abnormal inflammatory response and exert antioxidant activity which alleviates oxidative injury. | Tráj et al., 2023 |
| LUT decreases inflammation and oxidative stress in chronic obstructive pulmonary disease (COPD) via the NADPH oxidase 4-mediated NF-κB signaling pathway, which indicates that LUT may be employed to treat COPD. | Li et al., 2023 |
| LUT can diminish dexamethasone-induced necroptosis in bone microvascular endothelial cells via the RIPK1/RIPK3/MLKL pathway. This study provides new understanding of the mechanisms involved in the therapeutic activity of LUT on the glucocorticoid-induced osteonecrosis of the femoral head (GIONFH). Furthermore, suppressing necroptosis may become a future approach to treat GIONFH. | Xu et al., 2023 |
| LUT showed anticancer activity on high-grade serous ovarian cancer (HGSOC) cells by lowering vaccinia-related kinase 1 expression and activating the p53 signaling pathway, resulting in apoptosis and cell cycle arrest in G2/M as well as suppression of cell proliferation. In addition, LUT acted synergically with cisplatin both *in vivo* and *in vitro*. Hence, LUT could be employed in co-treatment on HGSOC. | Chang et al., 2023 |
| LUT exerts antidiabetic effects, by reverting hyperlipidemia, oxidative stress, and proinflammation in diabetic patients. Hence, LUT could be employed as an alternative for both management and treatment of diabetes. | Kahksha et al., 2023 |
| LUT acts as a potential mitigator of hepatic damage induced by aflatoxin B1 and may be employed in state-of-the-art treatments of human and animal hepatic illnesses. | Zaki et al., 2023 |
| LUT acts as an antiobesity and anti-inflammatory agent in a mouse model by lowering inflammation and muscle protein degradation, and as such proves to be a potentially effective treatment for obese sarcopenia. | Kim et al., 2023 |
| LUT suppresses the proliferation of stomach tumor cells and blocks the cell cycle in the S-phase. The mechanism by which it induces apoptosis in these cells is associated with the PI3K/AKT signaling pathway. | Yajie et al., 2023 |
| LUT ameliorates neutrophilic asthma via suppression of the IL-36γ secretion-mediated MAPK pathway. For this reason, LUT may be employed as a treatment for neutrophilic asthma. | Qiao et al., 2023 |
| LUT protects hepatocytes from palmitic acid-induced damage by improving antioxidant-mediated defence, which in turn alleviates ER stress and autophagy and induces autophagic flux. | Huang et al., 2023a |
| Both *in vitro* and *in vivo* systematic and transcriptomic analyses displayed the ability of LUT to ameliorate renal anemia resulted from renal fibrosis by acting on the sirtuin 1/forkhead box O3 pathway. | Li et al., 2022a |
| LUT possesses antiangiogenic activity and can also inhibit the proliferation and migration of cancerous cells *in vitro*. In addition, it is possible that LUT exerts its inhibitory activity through the PI3K/AKT signaling pathway. Hence, LUT may become a potential therapeutic agent to treat uveal melanomas characterized by high vascularization. | Chen et al., 2022 |
| LUT prevents the growth and migration/invasion of colon tumor cells via inhibition of the IL-6/signal transducer and activator of the transcription 3 signaling pathway. | Jiang et al., 2022 |
| LUT-induces vascular relaxation through myosin phosphatase reactivation and calcium desensitization. This mode of action seems to be associated with myosin phosphatase CPI-17 dephosphorylation by ROCK/PKC inhibition. | Yoon et al., 2023 |
| Low and medium doses of LUT can ameliorate β-cyfluthrin-mediated hepatotoxicity by alleviating peroxidative/nitrosative reactions and increasing total antioxidant capacity levels. | Mousavi et al., 2022 |
| LUT lowered the incidence of freezing behavior in mice with post-traumatic stress disorder (PTSD) in a situation-dependent manner, also displaying anti-depressant and anxiolytic activity. As such, LUT could become a potential tool to prevent traumatic stress such as PTSD. | Sur and Lee, 2022 |

| Key findings | Reference |
|---|---|
| LUT displayed antidepressant activity in a noise-induced depression-like mouse model, hence showing its potential as a novel treatment for chronic noise-induced depression. | Cheng et al., 2022 |
| LUT ameliorated hypoxia-induced pulmonary hypertension (HPH) in rats via a protective effect on pulmonary vascular endothelial function exerted by modulating the HIF-2α-Arg-NO axis and PI3K-AKT-eNOS-NO signaling pathway. The present study presents a possible novel approach to the treatment of HPH. | Ji et al., 2022 |
| LUT displayed a neuroprotective activity against hippocampal inflammation and autophagy caused by cerebral ischemia/reperfusion via activation of PPARγ in a rat model. | Li et al., 2022b |
| LUT can reverse tumor necrosis factor (TNF)-α-induced senescence. The results of this study indicate that LUT ameliorates TNF-α-induced inflammation and senescence of human nucleus pulposus cells via the Sirt6/NF-κB pathway. | Xie et al., 2022 |
| LUT exerts its anticancer activity by promoting HO-1 expression and inducing the labile iron pool to trigger ferroptosis in clear cell renal cell carcinoma (ccRCC). Hence, LUT may be an effective drug candidate for treating ccRCC. | Han et al., 2022 |
| LUT alleviates breast-cancer-related depression (BCRD) via modulation of the miR-124-3p/TNF-α/TNF receptor-associated factor 6-related pathway and suppression of neuronal cell pyroptosis and the ensuing inflammation. Hence, LUT could become a potential alternative treatment of BCRD. | Zhu et al., 2022 |
| The cardioprotective effects of LUT against myocardial ischemia-reperfusion injury (MIRI) are exerted on different signaling pathways: the main activities of LUT against MIRI are its anti-apoptotic, anti-oxidative and anti-inflammatory effects. | Pan et al., 2022 |
| LUT partially reverts multidrug resistance of osteosarcoma by upregulating PTN expression via miR-384 which leads to inhibition of the PTN/β-catenin/MDR1 axis. | Qin et al., 2022 |
| LUT inhibits cell proliferation, blocks the cell cycle, and causes DNA injury and apoptosis progression in colorectal cancer cells through modulation of the MAPK pathway. | Song et al., 2022 |
| LUT may effectively ameliorate cisplatin-induced cardiac injury and may become a potential drug candidate to treat chemotherapy-induced cardiovascular complications. | Qi et al., 2022 |
| LUT delayed oxaliplatin-induced tumor expansion by promoting apoptosis and suppressing heme oxygenase-1-mediated cytoprotection. These results highlight the potential of dietary LUT supplements in synergy with conventional chemotherapy to treat colorectal cancer. | Jang et al., 2022 |
| LUT suppresses neuroinflammation by preventing glial cell and NLRP3 inflammasome activation via regulation of p38 MAPK activity in the spinal dorsal horn, which leads to reduction of Lewis lung cancer-induced bone pain. | Zhou et al., 2022 |

***Acknowledgments***

***Conflict of interest***

The authors declare no conflict of interest.

## Notes

Priscilla Nadalin and Jae Kwang Kim contributed equally as first author.

## Declaration

### Acknowledgments

This research was supported by the Bio & Medical Technology Development Program of the National Research Foundation (NRF) and funded by the Korean government (MSIT) (No. 2022M3E5E6018649) and this work was also supported by the Institute of Information & Communications Technology Planning & Evaluation (IITP) grant funded by the Korea government (MSIT) (No.RS-202200155857, Artificial Intelligence Convergence Innovation Human Resources Development (Chungnam National University)).

### Conflict of interest

The authors declare no conflict of interest.

## Figures and Tables

**Table 1:**
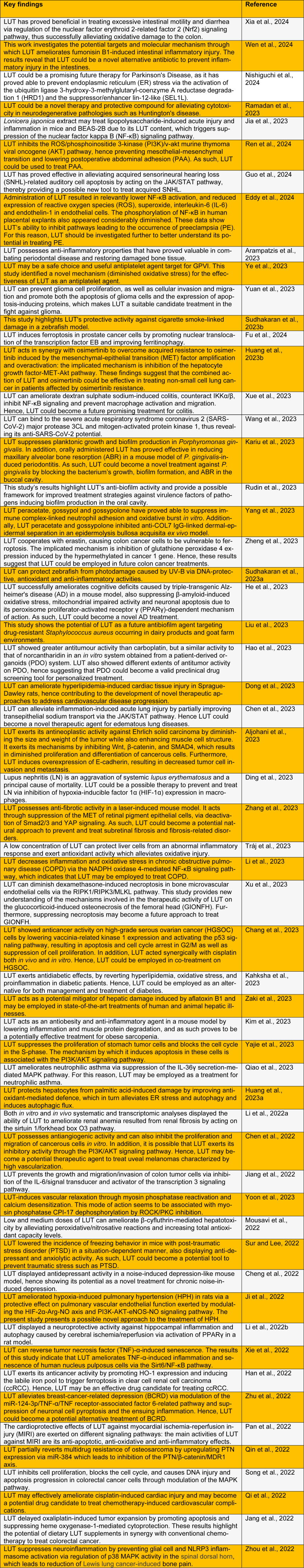
Recent studies on the biological and pharmacological activities of luteolin
